# The definition of neuronopathic Gaucher disease

**DOI:** 10.1002/jimd.12235

**Published:** 2020-04-03

**Authors:** Raphael Schiffmann, Jeff Sevigny, Arndt Rolfs, Elin Haf Davies, Ozlem Goker‐Alpan, Magy Abdelwahab, Ashok Vellodi, Eugen Mengel, Elena Lukina, Han‐Wook Yoo, Tanya Collin‐Histed, Aya Narita, Tama Dinur, Shoshana Revel‐Vilk, David Arkadir, Jeff Szer, Michael Wajnrajch, Uma Ramaswami, Ellen Sidransky, Aimee Donald, Ari Zimran

**Affiliations:** ^1^ Baylor Scott & White Research Institute Dallas Texas; ^2^ Prevail Therapeutics New York New York; ^3^ Centogene AG Rostock Germany; ^4^ Aparito, Ltd, Gwenfro Technology Park Wrexham UK; ^5^ Lysosomal and Rare Disorders Research and Treatment Center (LDRTC) Fairfax Virginia; ^6^ Department of Pediatric Hematology Cairo University Pediatric Hospital Cairo Egypt; ^7^ Bushey Herts WD23 1DU Bushey UK; ^8^ Clinical Science for LSD Hochheim Germany; ^9^ National Research Center for Hematology Moscow Russia; ^10^ Asan Medical Center, Department of Pediatrics Medical Genetics & Genomics Center Seoul South Korea; ^11^ International Gaucher Alliance Dursley UK; ^12^ Division of Child Neurology Institute of Neurological Science, Tottori University Faculty of Medicine Yonago Tottori Japan; ^13^ Gaucher Unit Shaare Zedek Medical Center Jerusalem Israel; ^14^ Hebrew University‐Hadassah Medical School Jerusalem Israel; ^15^ Clinical Haematology Peter MacCallum Cancer Centre and The Royal Melbourne Hospital Melbourne Australia; ^16^ Pfizer Inc., Clinical Professor of Pediatrics, New York University Langone School of Medicine New York New York; ^17^ Lysosomal Storage Disorder Unit Royal Free Hospital London UK; ^18^ Medical Genetics Branch, National Human Genome Research Institute National Institutes of Health Bethesda Maryland; ^19^ University of Manchester St Marys Hospital Manchester UK

**Keywords:** diagnosis, Gaucher disease, gaze palsy, lysosomal disease

## Abstract

Neuronopathic Gaucher disease (nGD) has a very wide clinical and genotypic spectrum. However, there is no consensus definition of nGD, including no description of how best to diagnostically separate the acute form—Gaucher type 2—from the subacute or chronic form—Gaucher type 3. In this article, we define the various forms of Gaucher disease with particular emphasis on the presence of gaze palsy in all patients with nGD. This consensus definition will help in both clinical diagnosis and appropriate patient recruitment to upcoming clinical trials.

## 1 INTRODUCTION

Gaucher disease is an inherited autosomal recessive lysosomal storage disease that is diagnosed in patients with both reduced activity of acid β‐glucosidase and mutations in the *GBA1* gene.^1^ Clinically, Gaucher disease is classified into three major forms based upon the absence or presence and rate of progression of neurological manifestations. The most common form in the Western world is Gaucher disease type 1 (GD1). It is distinguished by the lack of early onset central nervous system (CNS) involvement characteristic of the other two traditional clinical forms of neuronopathic Gaucher disease (nGD). The nGD forms are Gaucher disease type 2 (GD2) and Gaucher disease type 3 (GD3). GD2 is the acute neuronopathic form, which does not have an ethnic predilection and has an early onset CNS involvement, typically manifesting in the first 6 months of life and leading to death by age 2 years, although patients may live up to age 4 years or beyond with supportive medical care. GD3, or the chronic neuronopathic form, has a slightly later onset, CNS symptoms typically manifesting months to years after birth, and has a much slower neurological progression than is seen in GD2. Distinguishing GD2 from GD3 before age 2 years can be challenging in some cases.^2^ In non‐European countries such as Japan, China, Taiwan, Korea, India, Pakistan and Egypt, GD3 is the predominant form of Gaucher disease.^3^, ^4^, ^5^ In all three forms of Gaucher disease there is considerable phenotypic‐genotypic heterogeneity rendering it difficult to predict Gaucher type based solely upon genotype, with the exception that the presence of even one p.Asn409Ser allele (previously known as N370S) excludes the diagnosis of nGD.

Specific therapy for the non‐neurological (systemic) manifestations of GD1 and GD3 has been made possible by the introduction of enzyme replacement therapy (ERT) and more recently substrate reduction therapy (SRT) for GD1. However, these therapies do not penetrate the brain and therefore have no effect on the neurological aspects of GD2 or GD3. Consequently, treatment trials of novel therapies that cross the blood‐brain barrier, such as brain penetrant SRT, pharmacological chaperones, and intrathecal gene therapy are in development or in advanced planning stages.

Since only patients with GD2 or GD3 can be included in clinical trials for nGD, it is important to establish specific defining features of nGD. Currently, there is no agreement on a definition of nGD, other than one by exclusion (“the presence of neurological involvement in a patient with biochemically proven Gaucher disease, for which there is no explanation other than Gaucher disease”^6^) This lack of clarity has impeded the design of, and recruitment to, global patient registries and clinical trials. This is in part due to the broad spectrum of associated clinical phenotypes, and to the fact that phenotypes vary in different regions and ethnicities. Moreover, sometimes the defining oculomotor abnormality only manifests at later ages.

Our goal is to present a consensus definition of nGD by inclusion of criteria that will allow uniform patient recruitment to treatment trials and registries in different countries across the globe.

## 2 DEFINITION OF NGD

### 2.1 Required criteria

- Acid β‐glucosidase deficiency and bi‐allelic *GBA1* gene pathogenic variants.
- Gaze palsy, predominantly horizontal, with slow or absent saccades.^7^, ^8^ The gaze palsy is usually supranuclear but may progress to become nuclear. Supranuclear gaze palsy is a conjugate gaze limitation (slow saccades) that can be overcome by smooth pursuit or by the vestibulo‐ocular reflex (Video S1. There is often an associated limitation of abduction (likely due to sixth nerve paresis) with unilateral or more frequently bilateral esotropia (“convergent squint”). Smooth pursuit is often clinically normal (Video S2) but may become limited by sixth nerve paresis and may progress to complete gaze palsy in the most advanced disease stages, particularly in GD2 patients. Examination by a neuro‐ophthalmologist may be useful to identify gaze palsy in nGD.

Ascertaining the presence of gaze palsy is almost always feasible at the bedside and should be performed in a standardised manner (Video S1). Gaze palsy is also evident when the patient is asked to turn around rapidly, revealing an ocular “lag” (Video S3). Patients may blink or use head thrusts in an attempt to compensate for the dysfunctional saccadic mechanism. This is often the first observed manifestation of GD3 and can occur in early childhood before abnormal horizontal saccades are recognised.

#### 2.1.1 Clarification of terminology

The term oculomotor apraxia or saccadic initiation failure is often used to describe the gaze palsy in nGD. However, true gaze apraxia, also known as Cogan ocular motor apraxia, does not occur in nGD.^9^ It is characterised by inability or difficulty initiating a saccade, but when the saccade is initiated (eg, using head thrusts) it is of normal velocity. Difficulty initiating a saccade in nGD is always associated with a low saccade velocity. Therefore, these terms should not be used. The correct term is gaze palsy, either horizontal or vertical.

## 3 OTHER NEUROLOGICAL FEATURES THAT MAY BE PRESENT

- Gaze palsy may be an isolated finding in GD3 or coexist with other neurological abnormalities that might include widely variable cognitive impairment that develops in childhood with relative sparing of speech and language^10^; motor and coordination deficits such as ataxia; hyperreflexia without a pyramidal syndrome; tremor (mostly cerebellar or action tremor, dysmetria); partial complex or generalised seizures; stridor; dysphagia; dysarthria; dystonia; oppositional defiant behavioural abnormalities,^11^ and progressive myoclonic epilepsy.^12^ Rapid neurological decline does not occur in GD3 except in individuals with the progressive myoclonic epilepsy variant.

## 4 FEATURES SUGGESTIVE OF NGD

In the presence of enzymatic and genetic diagnosis of Gaucher disease, be alert to the possibility of late appearance of gaze palsy in a patient with neurological abnormalities described above.

- Background slowing on EEG, sometimes with epileptogenic activity, is often observed in nGD.
- Homozygosity of the p.Leu483Pro *GBA1* variant (previously known as L444P) is often associated with GD3.
- A number of non‐neurological clinical abnormalities are much more common in patients with GD3 on established ERT. These include: thoracic kyphosis with or without scoliosis with no evidence of vertebral body collapse; proliferation of lipid‐laden macrophages in a number of compartments “escaping” the effect of ERT (eg, Gaucheromas; mesenteric lymphadenopathy; infiltrative lung disease and vitreous opacities).
- Cardiac valvular and aortic calcifications, hydrocephalus, corneal opacity (only in p.Asp448His/p.Asp448His‐ previously known as D409H/D409H).

## 5 FEATURES THAT EXCLUDE THE DIAGNOSIS OF NGD

- Presence of p.Asn409Ser (previously known as N370S) variant.

## 6 DIAGNOSTIC FEATURES OF GD2

- Onset of neurological deficits by age 6 months* (in GD3 they appear at a later age).^13^
- Supranuclear gaze palsy/convergent squint*.
- Rapid neurological deterioration over months in the first 2 years of life*.
- Severe stridor and apnoea at some point, frequently necessitating tracheostomy by age 2 years*.
- Feeding difficulties due to abnormal swallowing often requiring tube feedings.^14^
- Development of spasticity, opisthotonus and/or progressive myoclonic epilepsy by age 2 years.
- Failure to achieve independent gait (GD3 always achieve independent gait).
- Death by age 4 years except when extreme life support measures are applied.
- Patients with neurological symptoms that straddle GD2 and GD3 exist.^2^
- GD2 can present in the perinatal period with hydrops fetalis or congenital ichthyosis.^13^

*Necessary and defining criteria.

## 7 ON THE OTHER HAND

- Common intercurrent neurological abnormalities in the absence of gaze palsy should not be considered nGD.
- Adult‐onset, isolated Parkinson disease, multiple system atrophy and dementia with Lewy bodies are not features of nGD. Rather, *GBA1* mutations are a *risk factor* for developing these neurodegenerative diseases.

We believe that efforts to evaluate patients in greater depth will aid our ability to refine such categorisation and phenotypic descriptions in the future using a combination of longitudinal clinical evaluations and genetic and demographic data to refine the broad spectrum of nGD currently described.

## CONFLICT OF INTEREST

Dr Schiffmann received research support and honorarium from Sanofi Genzyme and Protalix Biotherapeutics.

## INFORMED CONSENT

We obtained a written informed consent for the use of the eye movements videos.

## Supporting information


**Video S1** Supporting information

**Video S2** Supporting information

**Video S3** Supporting information
